# Revitalising Silver Nitrate for Caries Management

**DOI:** 10.3390/ijerph15010080

**Published:** 2018-01-06

**Authors:** Sherry Shiqian Gao, Irene Shuping Zhao, Steve Duffin, Duangporn Duangthip, Edward Chin Man Lo, Chun Hung Chu

**Affiliations:** 1 Faculty of Dentistry, The University of Hong Kong, Hong Kong, China; gao1204@hku.hk (S.S.G.); zhao110@hku.hk (I.S.Z.); dduang@hku.hk (D.D.); hrdplcm@hku.hk (E.C.M.L.); 2 General Dentist, Shoreview Dental, LLC, Keizer, 97303 OR, USA; steveduffin8@gmail.com

**Keywords:** silver nitrate, sodium fluoride, caries, early childhood caries, silver diamine fluoride

## Abstract

Silver nitrate has been adopted for medical use as a disinfectant for eye disease and burned wounds. In dentistry, it is an active ingredient of Howe’s solution used to prevent and arrest dental caries. While medical use of silver nitrate as a disinfectant became subsidiary with the discovery of antibiotics, its use in caries treatment also diminished with the use of fluoride in caries prevention. Since then, fluoride agents, particularly sodium fluoride, have gained popularity in caries prevention. However, caries is an infection caused by cariogenic bacteria, which demineralise enamel and dentine. Caries can progress and cause pulpal infection, but its progression can be halted through remineralisation. Sodium fluoride promotes remineralisation and silver nitrate has a profound antimicrobial effect. Hence, silver nitrate solution has been reintroduced for use with sodium fluoride varnish to arrest caries as a medical model strategy of caries management. Although the treatment permanently stains caries lesions black, this treatment protocol is simple, painless, non-invasive, and low-cost. It is well accepted by many clinicians and patients and therefore appears to be a promising strategy for caries control, particularly for young children, the elderly, and patients with severe caries risk or special needs.

## 1. Introduction

The use of silver in dentistry dates back to China in 659 A.D. It was used not only for its material properties, but also for the long-known antimicrobial effects. Silver has been adopted as an antimicrobial material for thousands of years. People in ancient Mexico used silver to make containers for storing water and food. A Roman pharmacopoeia written in 69 B.C. mentioned the use of silver as a disinfectant [1]. Silver nitrate is one of the most common silver salts and has antibacterial properties which have been widely used in medicine. Silver nitrate solution is a colourless and odourless solution. It has been used as a cauterizing agent in medicine for treating wounds, especially burned wounds [2]. Physicians used silver nitrate to chemically cauterize umbilical granulomas and warts. In the late 19th century, silver nitrate was used to treat venereal disease. A solution of 1% silver nitrate was used as eye drops for newborn babies to protect their eyes from the transmission of gonorrhoea from mothers during birth [3]. It has long been a common antimicrobial agent for medical use because of its broad spectrum of antibacterial activity, lack of bacterial resistance, and low toxicity [4]. However, the use of silver nitrate became subsidiary when penicillin and other antibiotics were introduced in the 1950s.

## 2. Antimicrobial Action of Silver Nitrate in Dentistry

Dentists often used silver nitrate as a disinfectant agent because of its escharotic, dehydrating, and sclerosing properties. Silver nitrate sticks were used to manage oral ulcers [5] and to reduce pain due to aphthous stomatitis [6]. Ammoniacal silver nitrate, or Howe’s solution, has been used as a sterilisation agent for disinfecting root canals [7]. Silver nitrate was used to sterilise disintegrated and infected dentine during restorative treatment [8]. It was also used for treating deep caries lesions and indirect pulp capping because the solution could permeate the affected dentine and fill the demineralised dentine with silver particles [9].

Silver nitrate’s antibacterial action could be related to silver’s extracellular and intracellular binding properties. Positive silver ions can electrostatically bind to the bacterial membrane and cell wall, which contain negative-charged peptidoglycans [10]. Additionally, a bacterial cell’s transport system may actively uptake silver ions. After silver ions are taken up, they can bind to various cellular structures such as proteins and deoxyribonucleic acid (DNA) [11]. When silver ions bind to sulfhydryl groups on enzymes, they will inactivate the enzymes, eventually resulting in the inactivation of the bacteria [12]. Silver ions can also bind to DNA. The binding will stabilise the helix of DNA and prevent its replication; hence, cell division will be hindered [13].

## 3. Silver Nitrate for Caries Management

Dental caries is a localised chemical dissolution of the tooth surfaces caused by metabolic events taking place in the biofilm covering the affected area over time [14]. Silver nitrate was adopted for caries management in the early 1900s based on the infectious character of dental caries and the disinfectant character of silver nitrate [15]. Five studies using silver nitrate solution to manage dental caries published between the 1940s and the 1970s were found through a literature search and are summarised in Table 1 [16,17,18,19,20]. Three of these studies examined the effectiveness of silver nitrate solution in preventing caries [16,17,19]. Their results showed no significant reduction in caries incidence compared with no treatment. Another two studies investigated the caries-arresting effect of silver nitrate solution [18,20]. They reported that silver nitrate could arrest dental caries on both permanent and primary teeth. Although the levels of evidence of these controlled trials are uncertain because the quality of reporting in randomised trials varied significantly in past studies, these studies still provide valuable information on the use of silver nitrate for caries control.

Apart from clinical trials, a laboratory study investigated the use of silver nitrate solution on artificial caries lesions in a microbial biofilm model [21]. The results showed that caries progression (lesion depth) was significantly lower with silver nitrate application when compared with no treatment. Silver nitrate has strong antibacterial action. Although some dentists continue to use silver nitrate for caries management, its dental use diminished when fluoride was introduced. In addition, the introduction of local anaesthesia—particularly lidocaine, which is safe and has a strong effect and scant allergenic action—also led to a shift from medical to surgical management of caries.

## 4. Medical Model in Caries Management 

Dental caries is a continuum of disease caused by bacterial infection, with patients at different degrees of risk, rather than just a lesion. Although most schools have taught the surgical model focusing on restoring the damage from caries in the past decades, the contemporary caries-management philosophy has changed from the traditional surgical approach to a medical model, which includes the use of fluoride therapy and antimicrobial agents [22]. Rather than focusing on restorative care, dentists should consider the bacterial infection, develop an effective strategy to treat the bacterial component of caries, and prevent further infectivity. Improvement of oral hygiene and diet change to decrease in sugar consumption are important. In addition, dentists should aim to remineralise rather than remove the caries (demineralised tissue), which can be facilitated with the use of fluoride agents.

## 5. Sodium Fluoride for Caries Management

Sodium fluoride is one of the most common fluoride agents used for promoting the remineralisation of enamel and dentine. Sodium fluoride varnish containing 2.26% (22,600 ppm) fluoride is known to be effective in caries prevention and has been used for this purpose for decades [23]. Sodium fluoride varnish is considered one of the most effective means of delivering fluoride for two reasons. First, it allows a high-concentration application of fluoride and hence minimises the amount to be ingested. Second, the varnish theoretically prolongs the contact time of the fluoride to the tooth surfaces. Thus, the slow release of fluoride can avoid the immediate loss of fluoride after application. The American Dental Association recommends that high-risk patients should receive fluoride varnish applications at 3- to 6-month intervals [24].

A systematic review and meta-analysis showed that a topical application of 5% sodium fluoride varnish remineralised early enamel caries and white spot lesions [25]. Although a complete understanding of the mechanism of fluoride action in dental caries is still being researched, it has been found that fluoride application on tooth surface produces calcium fluoride-like globules [26]. These globules are stabilised by protein phosphate and are fairly insoluble in the mouth. They act as a reservoir of fluoride at neutral acidity (pH). The dissolution rate of these globules increases when the pH is lowered during cariogenic challenges. Fluoride is released, which increases the saturation of calcium and phosphate ions in plaque fluid by lowering the solubility constant of the calcium and phosphate ions [26]. This helps to prevent the dissolution of calcium and phosphate from the tooth mineral and/or increases the rate of remineralsation or reprecipitation of the lost minerals. Fluoride can remineralise caries at early stages by forming fluorapatite and being incorporated into the demineralised enamel. However, a 30-month clinical trial reported that the application of 5% sodium fluoride varnish at 3-month intervals was not effective at arresting dentine caries [27].

Fluoride at a high concentration can exert its antimicrobial effects on biofilm through the inhibition of cellular enzymes in glycolysis and reduce acid production. Moreover, fluoride also enhances proton permeability of cell membranes, resulting in the acidification of cytoplasm and the inhibition of macromolecular synthesis [28]. A review concluded that fluoride has antimicrobial action, but the in vivo implications of this are still not clear [29].

## 6. Reintroducing the Use of Silver Nitrate Solution with Sodium Fluoride Varnish

Since silver nitrate has strong antibacterial action and sodium fluoride has good remineralising properties, a combined application of silver nitrate solution with sodium fluoride varnish has been introduced for managing dental caries. A study proposed the combined application of 25% silver nitrate solution followed by 5% sodium fluoride varnish as a non-invasive treatment of caries for children [30]. The study reported that more than 5000 children in the United States were treated with this protocol. The results showed that almost all caries lesions were arrested after treatment. Radiographic examination showed the formation of a radio-opaque layer, suggesting the generation of secondary dentine [30]. Subsequent restorations could be done without the need for local anaesthesia, which also suggested that secondary dentine was formed after the silver nitrate and sodium fluoride were applied. The clinic followed up the treated teeth in these children and found that almost all (98%) of the caries lesions from a random sample of 106 children remained arrested for up to 4 years after treatment [30]. After the initial study, whenever the dentist found a cavitated caries lesion, silver nitrate solution followed by sodium fluoride varnish were used after explaining the procedure and obtaining patient consent. No significant adverse effect was found to be associated with this non-invasive treatment. As more caries were treated with this protocol, fewer children required conventional treatment under general anaesthesia. The study reported that 26 cases were admitted for in-hospital restorations in 2008, but no patient required operating room treatment by 2011. Last but not least, the patient response was very gratifying. Patient apprehension due to previous unfavourable dental experiences decreased significantly. In addition, other family members, including young children who watched the treatment, became less apprehensive towards their subsequent dental care [30].

**Figure 1:**
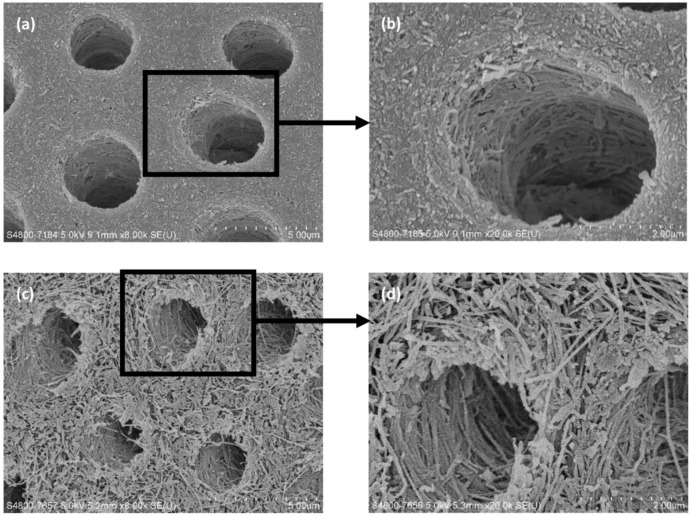
Scanning electron microscopy images of artificial dentine caries with and without topical application of 25% silver nitrate solution followed by 5% sodium fluoride varnish: (**a**) 8000× and (**b**) 20,000× magnification views of the group treated with silver nitrate and sodium fluoride; (**c**) 8000× and (**d**) 20,000× magnification views of the group treated with deionised water.

A recent laboratory study investigated the caries-arresting effect of 25% silver nitrate solution followed by 5% sodium fluoride varnish on artificial dentine caries [31]. The results of X-ray microtomography and spectrophotometry with hydroxyproline assay showed that the treatment could remineralise the artificial caries and prevent the degradation of dentine collagen. Scanning electron microscopy also confirmed the remineralisation of the artificial caries, and the collagen fibres were no longer exposed after treatment (Figure 1).

**Figure 2:**
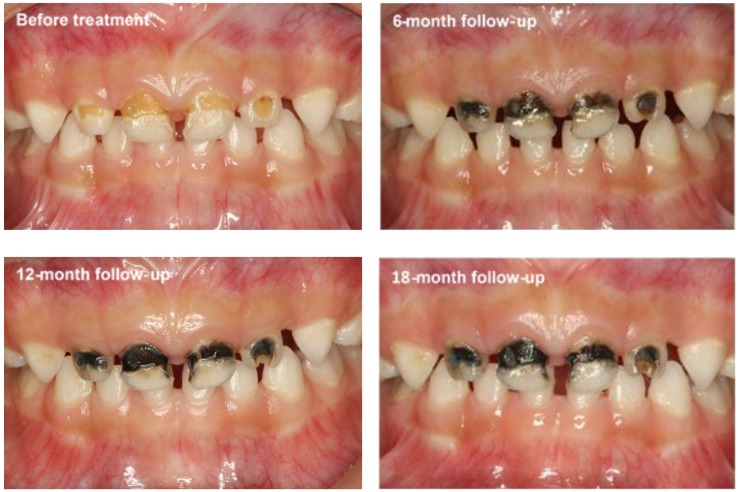
Dental caries before and after the application of 25% silver nitrate solution followed by 5% sodium fluoride varnish.

A double-blind randomised clinical trial was performed to compare the caries-arresting effect of the silver nitrate solution followed by sodium fluoride varnish protocol with that of silver diamine fluoride (SDF) [32]. More than 1000 kindergarten children aged 3–4 years with cavitated caries lesions were treated with the protocol or SDF (positive control) every six months. The 12-month results found no difference in caries-arresting effects between using the silver nitrate solution followed by sodium fluoride varnish protocol and using SDF [33]. Arrested caries lesions were stained black (Figure 2). No other significant side effect was observed. The study also found that the caries-arresting rate was influenced by the child’s oral hygiene and the location of the caries surface.

## 7. Advantages and Disadvantages

Although few studies report on the use of silver nitrate solution followed by sodium fluoride varnish protocol for treating caries, the available results suggested that the protocol was effective in arresting dental caries. More clinical trials are necessary to generate more evidence. However, clinicians may use this simple, low-cost, and non-invasive protocol to control pain and infection due to caries progression. The treatment can be carried out in a simple clinical setting with basic instruments. This protocol does not require expensive or complicated equipment or support infrastructure, such as piped water and electricity. Para-dental staff such as dental hygienists and therapists can be trained to provide the treatment under a dentist’s supervision. Because of the low cost of the material and the relatively short time required, this protocol should be affordable in most communities. Last but not least, this treatment protocol is non-invasive, and the risk of cross-infection is low.

A temporary henna-appearing stain will appear when silver nitrate solution comes into contact with skin. The skin pigmentation is temporary and will disappear within one to two weeks because the silver does not penetrate into the dermis. A permanent stain will occur when silver nitrate comes into contact with other objects such as clothing and counters. In addition, unintentional placement of a high concentration of silver nitrate to the cornea may lead to blindness because the solution can burn or opacify the cornea. Thus, the patient must wear protective eye glasses. The operator should wear personal protective equipment, keep the solution in small quantities, and pay attention when handling silver nitrate solution during the application.

The inherent disadvantage of using silver nitrate followed by sodium fluoride varnish to arrest caries is that the caries lesions will be permanently stained black. Hence, it is very important to explain and to inform the patient before treatment to avoid patient dissatisfaction. A survey in the United States found that staining on children’s posterior teeth was more acceptable to parents than staining on anterior teeth after caries treatment [34]. Although most of the parents had aesthetic concerns about the staining of anterior teeth, many of them would still accept the non-invasive treatment for their children over conventional restorative care under general anaesthesia. In addition, many parents tended to compromise on the aesthetics issue because of their children’s uncooperative behaviour regarding the traditional treatment. To address the aesthetic issue, some clinicians have suggested placing a tooth-coloured material such as glass ionomer cement on the stained and arrested caries lesion.

## 8. Safety Issues

A review concluded that very limited data is available on the potential toxic effects of silver, although it does accumulate in some human tissues and organs [35]. In addition, there are few known reports of silver allergy. Instead, silver is incorporated into various medical devices such as catheters and bone cement because of its low cytotoxicity.

Silver nitrate solution is toxic and corrosive. It is regarded as a poisonous chemical which can cause burns. However, exposure to a small amount of the solution will not produce immediate or even any side effects other than temporary henna-appearing staining of the skin. Complications and side effects can become more noticeable with repeated exposures. The toxicity of silver nitrate is related to the dosage. Ingestion of large quantities of silver nitrate (more than 2 g) can be fatal because silver nitrate rapidly reacts with chloride to precipitate highly insoluble silver chloride, which will lead to a fatal electrolyte imbalance. However, the dosage of silver nitrate used for treating dental caries is extremely small. A 25% silver nitrate solution is commercially available (Gordon Laboratories, Upper Darby, PA, USA). One millilitre of 25% silver nitrate solution contains 0.25 g silver nitrate and comprises 20 drops of the solution (according to the drop defined by the metric system). A single drop of the solution contains 13 mg silver nitrate and is equivalent to 0.33% of a fatal dose. One drop of the solution can serve approximately 20 applications to tooth surfaces using a micro-brush.

Some dentists are concerned about the cytotoxicity of silver nitrate to the pulp tissue because of its superior penetration capability. Weiss reported the histological effects of silver nitrate solution on caries teeth [36]. Silver nitrate was observed to penetrate the dentine tubules under the cavity almost to the pulp. Another study examined the pulpal response when silver nitrate was directly and indirectly applied onto the pulp tissue [37]. Silver particles were identified in the non-exposed pulp after the silver nitrate application. Moreover, inflammatory changes were found within the pulp beneath the treated lesion. Large globules of free silver were found in the blood clot after silver nitrate was applied to the exposed vital pulp tissue. A broad inflammatory zone of pulp immediately under the blood clot was observed. Nevertheless, this reaction could prevent the further penetration of silver nitrate into the rest of normal pulp tissue.

Some researchers have considered silver nitrate application to have no significant cytotoxic effect on the pulp if an adequate amount of sound dentine exists between the caries lesion and the pulp [38]. In addition, the time of application can influence the depth of penetration of silver nitrate in a caries lesion. A study reported that silver nitrate penetrated to an average of 0.3 mm through caries dentine after a 1-min application. The penetration increased to 0.7 mm after a 3-min application and 1.3 mm after a 10-min application [38]. Dentists should make judgements on the time of application for silver nitrate solution in clinical care based on the caries lesion’s activity.

## 9. Clinical Applications

Silver nitrate solution and sodium fluoride varnish are readily available in most countries. The application of silver nitrate solution followed by sodium fluoride varnish protocol is simple, non-invasive, and low-cost. As traditional dental treatment is often not affordable or available in many communities, this silver nitrate solution and sodium fluoride varnish protocol can be a promising alternative strategy for managing caries. Various concentrations of silver nitrate are available, and further study should be performed to find out the optimal concentration in caries management. The protocol is particularly valuable for young children, elderly populations, and people with special needs. Early childhood caries is globally prevalent among children and remains a challenging issue worldwide. It confers significant health and quality-of-life impacts to children and their families. Because of uncooperative behaviour, traditional restorative treatments in young children often require advanced clinical equipment and pharmacological behaviour-guidance modalities (i.e., sedation and general anaesthesia). Moreover, the cost of treating severe early childhood caries is high, especially when hospitalisation is necessary. Thus, conventional management for dental caries is often neither accessible nor affordable for young children, particularly for children from disadvantaged families. According to a retrospective study in the United States, the treatment cost of silver nitrate solution and sodium fluoride varnish was lower than that of the conventional treatment for dental caries [39].

Many elderly people have high risk of caries, especially root caries, which is very challenging to restore. The World Health Organisation recommended that governments adopt strategies to improve the oral health of elderly populations [40]. Silver nitrate solution and sodium fluoride varnish can be a viable option for managing dental caries among elderly populations. Moreover, people with special management considerations or special health care needs may also benefit from this protocol for caries management [41].

## 10. Conclusions

In conclusion, studies have suggested that a combined application of silver nitrate solution followed by sodium fluoride varnish can be used to arrest dental caries. The treatment protocol is simple, non-invasive, painless, and low-cost. It can be a promising strategy for treating dental caries among young children, elderly populations, and people with special needs. As there are limited studies in the literature about this treatment, more randomised clinical trials should be conducted to provide stronger evidence for using silver nitrate solution followed by sodium fluoride varnish.

## Figures and Tables

**Table 1:** Clinical trials of silver nitrate for caries management.

Author(s)Year [Ref.]	Aim	Study Design	Main Findings
Klein et al., 1942 [16]	Caries prevention	Permanent teeth (*n* = 92)	Caries incidence:
		Follow-up duration: 5 years	Group 1—79%
		Group 1—AgNO_3_ one-off application (*n* = 37)	Group 2—77%
		Group 2—No treatment (*n* = 55)	*p* = 0.821
James et al., 1954 [17]	Caries prevention	Permanent teeth (*n* = 248)	Caries incidence:
		Follow-up duration: 2 years	Group 1—69%
		Group 1—AgNO_3_ one-off application (*n* = 124)	Group 2—67%
		Group 2—No treatment (*n* = 124)	*p* = 0.683
Miller et al., 1959 [19]	Caries prevention	Permanent teeth (*n* = 129)	Caries incidence:
		Follow-up duration: 2 years	Group 1—72%
		Group 1—AgNO_3_, twice-monthly application (*n* = 67)	Group 2—73%
		Group 2—No treatment (*n* = 62)	*p* = 0.905
Schultz-Haudt et al., 1956 [18]	Caries arrest	Primary teeth (*n* = 229)	Caries arresting rate:
		Follow-up duration: 1 year	Group 1—82%
		Group 1—Caries removal + AgNO_3_ application (*n* = 136)	Group 2—17%
		Group 2—Caries removal only (*n* = 93)	*p* < 0.001
Hyde, 1973 [20]	Caries arrest	Permanent teeth (*n* = 196)	Caries arresting rate:
		Follow-up duration: 2 years	Group 1—31%
		Group 1—AgNO_3_ one-off application (*n* = 96)	Group 2—18%
		Group 2—No treatment (*n* = 100)	*p* = 0.031
